# A methodological protocol for multimodal profiling of conversational abilities in mandarin-speaking children with and without developmental language disorder

**DOI:** 10.3389/fpsyg.2026.1704308

**Published:** 2026-05-05

**Authors:** Cai Wang, Jiamin Xu, Mengmeng Yao, Jingyu Wang, Qin Hong, Hao Zhao, Xia Chi

**Affiliations:** 1 Department of Child Health Care, Women’s Hospital of Nanjing Medical University (Nanjing Women and Children’s Healthcare Hospital), Nanjing, Jiangsu, China; 2 Nanjing Medical Key Laboratory of Developmental Behavioral Pediatrics, Nanjing, Jiangsu, China; 3 Research Center for Special Operations and Rehabilitation Robotics, Nanjing Institute of Technology, Nanjing, Jiangsu, China

**Keywords:** child, communication, language disorders, pragmatics, preschool

## Abstract

**Introduction:**

Developmental Language Disorder (DLD) is a common neurodevelopmental condition that affects both the structural and pragmatic aspects of language. Conversational abilities—including topic initiation, maintenance, repair, turn-taking, and the integration of non-verbal behaviors—are essential for social communication and peer relationships. While a substantial body of research has described conversational difficulties in DLD among English-speaking populations, there is limited evidence on how these impairments manifest in Mandarin-speaking children. There is a critical need for culturally adapted assessment approaches; thus, this protocol primarily aims to evaluate the feasibility of a multimodal framework tailored specifically to the Mandarin-speaking context.

**Methods:**

This protocol describes an age-stratified cross-sectional observational study with two phases. In Phase 1, an adapted multimodal profiling framework will be developed and piloted through semi-structured free conversation and role-play tasks, recorded with audio–video equipment. Transcription will follow Codes for the Human Analysis of Transcripts (CHAT) conventions within European Distributed Corpora (EUDICO) Linguistic Annotator (ELAN), with word segmentation supported by Jieba and manual correction. Annotation tiers will capture utterance-level features, communicative acts (Inventory of Communicative Acts-Abridged [INCA-A]), repairs, turn-taking, and gesture–speech alignment (stroke apex ±500 ms). Feasibility (session completion, recording length, and utterance yield) and reliability (Cohen’s *κ*, ICC, and drift checks) will be evaluated to refine the coding manual. In Phase 2, the finalized protocol will be applied to 90 children with DLD and 90 typically developing (TD) peers (aged 4–6, matched for age and sex). Data analysis will quantify primary outcome measures (topic maintenance ratio, successful repair rate, gesture–speech synchrony index) and secondary outcome measures (lexical diversity, initiation–maintenance balance, and turn-taking productivity), with exploratory analyses of demographic and home-environment moderators.

**Discussion:**

This protocol advances methodological research by providing a transparent and reproducible framework for multimodal profiling of pragmatic language in Mandarin-speaking preschoolers. Its strengths include multimodal data integration and explicit feasibility benchmarks. Limitations include the cross-sectional design and a modest sample size. Future research may extend the protocol to longitudinal studies, contributing to the establishment of standardized procedural benchmarks and improved data comparability within this specific linguistic environment.

## 1 Introduction

Developmental Language Disorder (DLD) is a prevalent neurodevelopmental condition affecting approximately 7–10% of children, and is characterized by persistent difficulties in acquiring and using language across modalities and contexts, despite normal hearing, non-verbal intelligence, and an absence of neurological or emotional disorders (Bishop et al., 2017; Befi-Lopes et al., 2024; Liang et al., 2025). In addition to delays in structural language domains such as phonology, vocabulary and syntax, mounting evidence highlights the challenges children with DLD face in pragmatic language—defined as the ability to use language effectively in social contexts to achieve communicative goals (Befi-Lopes et al., 2024). Crucially, conversational abilities, including initiating, maintaining, repairing discourse and effectively integrating non-verbal cues such as gesture and gaze, form a central aspect of pragmatic competence and directly influence children’s peer relationships and educational participation (Landa, 2005; Loukusa et al., 2018; Trujillo et al., 2018).

Building on this, conversational abilities can be further specified as the set of skills enabling children to engage in reciprocal verbal exchanges, including topic initiation, topic maintenance, conversational repair and turn-taking (Airenti, 2017). These behaviors are not only indicative of a child’s linguistic competence, but also of their social cognition and executive functioning (Bishop et al., 2000). Importantly, effective conversation relies not solely on verbal utterances but also on non-verbal modalities—gestures, for example—which serve to supplement, clarify, or even replace spoken language during interaction (Goldin-Meadow, 2004; Saubesty and Tellier, 2015). Gestures can help regulate turn-taking, emphasize discourse focus and scaffold meaning construction, especially in early childhood or in populations with language difficulties (Giberga et al., 2025). Some evidence suggests that deficits in conversational abilities—such as initiating and maintaining dialogue, responding flexibly to conversational partners and managing the flow of interaction—can negatively impact children’s social functioning. Such limitations may hinder their capacity to form and maintain peer relationships, negotiate social play and participate effectively in classroom settings (Bauminger-Zviely et al., 2014).

A substantial body of research in English-speaking populations has documented conversational impairments in DLD. For example, Abbot-Smith et al. reported that children with DLD are much more likely to give minimal responses—such as brief acknowledgments or single-word answers—which undermines their ability to maintain conversational topics and engage in sustained dialogue with peers (Abbot-Smith et al., 2023). van Balkom et al. observed that children with DLD exhibit marked difficulties with turn-taking, topic initiation and topic maintenance in naturalistic interactions, often relying on restricted linguistic forms and non-verbal strategies, while contributing fewer verbal turns than typically developing children (Abbot-Smith et al., 2023). While some evidence suggests that children with DLD may rely heavily on simple deictic gestures (e.g., pointing) (Lee and Lee, 2015), recent findings highlight a more complex multimodal profile: specifically, these children tend to produce significantly higher rates of supplementary gestures—conveying semantic content absent from their speech—as a strategy to compensate for limited expressive vocabulary and achieve greater communicative complexity (Rombouts et al., 2023). Barachetti and Lavelli found that during shared book reading, children with DLD frequently produced problematic or incomplete responses, requiring their mothers to provide significantly more high-supportive repairs to achieve minimally acceptable answers—highlighting these children’s limited capacity for conversational repair and their reliance on adult scaffolding to sustain interaction (Barachetti and Lavelli, 2011). Taken together, these findings underline the importance of using ecologically valid and culturally appropriate approaches to better understand conversational difficulties in children with DLD.

While pragmatic deficits have been extensively studied in English-speaking contexts, little is known about their manifestation in Mandarin-speaking children, where distinct linguistic and cultural norms may uniquely shape conversational behavior. Mandarin Chinese is a topic-prominent, high-context language characterized by frequent ellipsis, pronoun dropping and indirectness, where meaning heavily depends on context and non-verbal modalities such as gestures, facial expressions and prosody (Ladha et al., 2018; Han, 2019). These typological and cultural features suggest the necessity for culturally adapted and ecologically valid assessments rather than directly adopting methods developed for English-speaking populations.

Observing children’s interactions in familiar and naturalistic settings, such as free play or narrative tasks, is widely regarded as the most effective approach to studying conversational abilities (Lieven and Stoll, 2013; Gökce, 2019). Language sample analysis (LSA) conducted in these settings enables the collection of spontaneous language data that closely reflects authentic communicative behaviors and reduces the performance bias associated with standardized tests (Eisenberg and Guo, 2016; Bawayan and Brown, 2022). LSA allows for the simultaneous examination of multiple facets of expressive language—including pragmatic use and language output metrics such as total number of utterances, mean length of utterance (MLU), type–token ratio (TTR) and D-value—thus providing a comprehensive and ecologically valid profile of children’s conversational performance (Yang et al., 2022). Additionally, multimodal annotation tools such as the Child Language Data Exchange System (CHILDES) CHAT transcription system, Computerized Language Analysis (CLAN) software, and the EUDICO Linguistic Annotator (ELAN) platform enable detailed analysis of gesture–speech integration, offering a more nuanced understanding of pragmatic functioning (Kong et al., 2015; Sanchez et al., 2019; Fromm et al., 2020).

While various systems have been developed to capture children’s communication, they often exhibit specific limitations in temporal precision and linguistic adaptation. For instance, the CHILDES (CHAT/CLAN) system remains the gold standard for language sampling; however, its architecture is predominantly verbal-centric, often treating non-verbal behaviors as secondary, unstructured “comments” rather than synchronized and codable streams (MacWhinney and Snow, 1990). While McNeill’s (1994) gesture framework is foundational and provides rich semantic categories, it was primarily designed for low-context Western languages and lacks standardized temporal synchronization benchmarks (e.g., specific millisecond windows) required to identify subtle coordination deficits in DLD. Furthermore, established tools such as Systematic Analysis of Language Transcripts (SALT) and Language Environment Analysis (LENA) focus primarily on verbal metrics and acoustic-environmental data, respectively. Both systems lack an integrated architecture to capture non-verbal modalities, particularly the fluid coordination between speech and gestures (Colombani et al., 2022; Almubark et al., 2023). Consequently, there is a clear need for a unified protocol that integrates precise temporal standards with culturally adapted pragmatic categories for Mandarin-speaking preschoolers, and the feasibility of which remains to be validated within this unique linguistic context.

In light of these limitations, several critical gaps remain unaddressed. First, although general frameworks exist, there is a lack of data on Mandarin-speaking populations, whose conversational behavior is shaped by a high-context, topic-prominent linguistic structure. Second, the multimodal aspect of communication—specifically the coordination between gesture and speech—remains under-researched in children with DLD. Finally, there is a need for more ecologically valid assessment methods that move beyond standardized testing to analyze spontaneous interaction in naturalistic settings.

To bridge these methodological gaps, the primary objective of this protocol is to validate the feasibility and suitability of a multimodal framework specifically adapted for Mandarin-speaking preschoolers. This protocol advances the field through three primary contributions: first, it provides a tailored methodological adaptation for Mandarin-speaking preschoolers by refining coding categories to capture these unique linguistic features. Second, it introduces a standardized ±500 ms synchronization window for gesture–speech alignment, providing a reproducible technical benchmark that was previously underspecified in the literature. Third, it offers an integrated coding architecture that captures the functional synergy between verbal and non-verbal channels. Specifically, we hypothesize that children with DLD will demonstrate a distinctive multimodal profile compared to TD peers, characterized by: (1) reduced coordination, with lower gesture–speech synchrony and topic maintenance ratios; (2) diminished conversational productivity, marked by longer response latencies and lower lexical diversity; and (3) restricted communicative acts with a greater reliance on adults. By validating this cross-linguistic adaptation, this research aims to provide a methodological tool to advance the theoretical understanding and clinical assessment of pragmatic competence in Mandarin-speaking children. Ultimately, these findings will inform the development of contextually appropriate and culturally sensitive diagnostic and intervention tools.

## 2 Methods

### 2.1 Objectives of the study

The primary objective of this study is to propose and validate the feasibility and suitability of a standardized, multimodal methodological protocol for profiling conversational abilities in Mandarin-speaking children with and without Developmental Language Disorder (DLD), providing a structured framework for subsequent empirical investigations. Specifically, the objectives are:

(1) To formulate a reproducible multimodal profiling method for conversational abilities in preschool children, with operationalized tasks, annotation rules, coding procedures and clearly defined measures.
(2) To pilot and refine the method in Mandarin-speaking children to establish feasibility and inter-rater reliability.
(3) To apply the finalized method to describe and compare conversational characteristics in DLD vs. typically developing peers within a Mandarin-speaking context.

### 2.2 Primary and secondary outcome measures

#### 2.2.1 Primary outcome measures

Core pragmatic indicators: Topic maintenance ratio (proportion of contingent turns within topic episodes), successful repair rate (proportion of repair attempts resolving the breakdown within two subsequent turns) and gesture–speech synchrony index (proportion of lexical affiliates accompanied by gesture stroke apex within ±500 ms).

#### 2.2.2 Secondary outcome measures

(1) Lexical diversity: type–token ratio (TTR), Vocabulary Diversity – D (VOCD-D), and Measure of Textual Lexical Diversity (MTLD).
(2) Initiation and maintenance balance: frequency of child-initiated turns vs. adult-elicited responses, and the initiation-response ratio as an index of conversational proactivity.
(3) Distribution of communicative acts: categorization of child utterances according to INCA-A, and relative frequency of each communicative act type.
(4) Repairs: frequency and type of repair attempts (self-repair and other-initiated repair), and success rate of repairs (resolution within the next two turns).
(5) Turn-taking productivity: number and proportion of child turns; mean length of utterance (MLU); average number of words per turn; latency between partner turn and child response.

#### 2.2.3 Exploratory measures

Exploratory analyses will examine associations between conversational measures and demographic or home environment factors, and will contrast DLD with TD peers to identify distinctive pragmatic features.

### 2.3 Patient and public involvement

Patients or the public are not involved in the formal design, conduct, reporting or dissemination plans of this protocol. However, the elicitation tasks will be piloted with preschool children and their parents to ensure feasibility, ecological validity and child engagement. Parents will provide feedback on task acceptability and recording procedures, which will inform refinements to the final protocol. Teachers and clinicians from participating kindergartens will also advise on task selection to ensure that the materials are developmentally appropriate and culturally familiar. To ensure ethical reciprocity and voluntary participation without monetary inducement, each child will receive a small, age-appropriate gift (e.g., a picture book) upon completing the tasks. Furthermore, families of children with DLD will be provided with an individualized clinical feedback report—summarizing results from their child’s language, non-verbal intelligence and conversational assessments—to support their ongoing intervention and clinical management.

### 2.4 Study design

This study is a prospective, cross-sectional observational design that consists of two sequential phases: (1) method development and piloting, and (2) application of the method in DLD and TD groups. The design ensures that the multimodal profiling method is first established and refined, then systematically applied to describe and compare conversational characteristics in both Mandarin-speaking preschool children with DLD and their typically developing (TD) peers.

#### 2.4.1 Phase 1: method development and piloting

In the first phase, the goal is to establish a reproducible, multimodal coding and analysis framework for conversational abilities. These tasks will provide the raw material for piloting the coding system.

##### 2.4.1.1 Elicitation tasks

The purpose of the elicitation procedures is to obtain an ecologically valid conversational language sample of sufficient length and variability to permit reliable analysis of pragmatic and multimodal features. Each child will contribute a combined sample of approximately 20–30 min, drawn from multiple task contexts, to ensure that at least 20 min of analyzable data and at least 100 child turns are available for transcription and coding.

To ensure the representativeness of these samples, particularly for children with DLD who may exhibit task disengagement, the examiner follows a naturalistic interaction style characterized by low clinician control and high responsivity (Leadholm and Miller, 1994). In cases where a child deviates from the elicitation context—such as shifting to unrelated topics or disengaging from the role-play—a structured redirection hierarchy is employed. This includes utilizing facilitation techniques such as 5 s intentional pauses, parallel talk to describe the child’s current focus and low-constraint open-ended prompts to gently guide the interaction back to the task (Leadholm and Miller, 1994). These deviations are explicitly coded within the transcript rather than excluded, thereby preserving the ecological validity of the child’s spontaneous pragmatic profile.

The rationale for this dual-task design—incorporating both free conversation and semi-structured role-play—is to capture a broad spectrum of communicative behaviors across different interactional contexts. Specifically, the free conversation task is employed to assess general social-pragmatic initiation and topic maintenance in a naturalistic flow. In contrast, the semi-structured role-play (e.g., “Going Shopping”) is specifically designed to elicit functional communicative acts—such as requests, protests and negotiation—that might not occur as frequently or consistently in open-ended dialogue. By combining these complementary contexts, the protocol ensures a comprehensive profile of the child’s pragmatic competence while maintaining high ecological validity.

###### 2.4.1.1.1 Semi-structured free conversation

The first task is a semi-structured free conversation in which the researcher engages the child in open-ended dialogue supported by a puppet dog as a conversational prop. Topics are selected from the child’s everyday experiences, such as birthdays, pets, favorite games, toys or cartoons (see Supplementary File 1). The aim of this task is to elicit spontaneous verbal expression, provide opportunities for topic initiation, and maintain conversational flow. Each session is expected to last approximately 10–15 min and to yield a sufficient number of child utterances for subsequent analysis.

###### 2.4.1.1.2 Role-play activity

The second task is a role-play activity that simulates a supermarket shopping experience (see Supplementary File 1). In the first phase of the activity, the child takes the role of a customer while the researcher plays the shopkeeper. In the second phase, the roles are reversed: the child acts as the shopkeeper and the researcher as the customer. This structured yet playful context is designed to provide ecologically valid opportunities for conversational turn-taking, clarification requests, repair attempts and multimodal behaviors such as gestures accompanying speech. Each role-play interaction will also last approximately 10–15 min, thereby complementing the spontaneous exchanges observed in the free conversation task.

##### 2.4.1.2 Video recording environment and equipment

All data collection will take place in a quiet, distraction-free room within the kindergarten or clinic setting to minimize noise interference and ensure high-quality recordings. The room will be arranged with stable lighting that allows clear visibility of the child’s face and upper body, enabling accurate gesture analysis. A fixed-position high-definition camera will be placed in front of the child, with a lateral view provided, when possible, to capture both verbal and non-verbal behaviors. High-quality audio will be recorded using a professional digital recorder, such as the SONY PCM-100. Each session will be designed to produce at least 20 min of analyzable conversation and at least 100 child utterances, which are required for inclusion in the pilot dataset.

##### 2.4.1.3 Transcription

For each child, a dedicated ELAN file will be created, and the corresponding audio and video recordings will be imported. A speech tier will be established to annotate utterances, and transcription will be performed at the utterance level with verbatim representation of all spoken language, including fillers, repetitions and incomplete words. Preserving these elements is essential for later coding of pragmatic and repair phenomena. To maintain comparability across participants, transcription will be limited to the first 20 min of each recording.

Utterance boundaries will be determined using a combination of syntactic and prosodic cues, with pauses longer than 2 s marking a new utterance. Overlapping speech between the child and the conversational partner will be transcribed on separate aligned tiers, and non-verbal vocalizations such as laughter or sighs will be annotated to capture their pragmatic role.

To prepare transcripts for quantitative analysis, each utterance will be segmented into words using the Jieba word segmentation module in Python as a first-pass and automated tool. The automated output will be exported into plain-text format, with segmented words separated by spaces. Trained coders will manually review and correct all segmentation results to ensure accuracy, particularly in cases involving child-specific speech errors or unconventional phrasing. Based on prior experience, this workflow typically requires approximately 5 min of manual correction for a 20-min conversational sample, significantly improving processing efficiency while maintaining linguistic precision.

For quality assurance, at least 20% of transcripts will be double-checked by a second transcriber, and inter-rater reliability for utterance segmentation will be calculated (target *κ* ≥ 0.75). Discrepancies will be resolved through consensus discussion, and emerging issues will be incorporated into the evolving transcription manual. Drift control will be implemented by re-checking a subset of previously transcribed samples after every 10 participants.

All finalized transcripts will be stored in CHAT-compatible text format and linked to the corresponding ELAN annotation files through unique session identifiers. This ensures that verbal data can later be synchronized with gesture coding during the multimodal annotation process, forming the basis for method validation in Phase 1 before application to larger groups in Phase 2.

##### 2.4.1.4 Coding framework

All conversational samples will be annotated in ELAN using a multimodal framework that organizes coding into distinct layers: lexical, pragmatic, repair, turn-taking, gestural, and multimodal coordination. Each layer captures observable behaviors and supports the derivation of conversational measures. Table 1 summarizes the coding categories and their associated analytic indices.

**Table 1:** Coding categories and descriptions for conversational data.

Domain	Coding or derived categories	Operational definition
Lexical diversity	TTR	Type–token ratio: ratio of the number of unique word types to the total number of words in the sample.
	VOCD-D	Estimated by curve fitting the relationship between TTR and sample size, using random sampling to model how vocabulary diversity changes with text length.
	MTLD	Total number of words in the sample divided by the number of factors, where a factor is the sequence length required for TTR to drop below a preset threshold.
Communicative acts	INCA-A	Categories based on the Inventory of Communicative Acts-Abridged (INCA-A), covering communicative intentions and speech acts (see Supplementary File 2).
	Distribution of communicative acts	Proportion of utterances in each INCA-A category.
Initiation and maintenance	Initiation types	Initiation Verbal-Directed (IVD): Starting a topic using only verbal language. Initiation Non-Verbal-Directed (IND): Using only non-verbal means (e.g., head movements, gestures and eye contact). Initiation Verbal with Non-Verbal (IVN): Using a combination of verbal and non-verbal means.
	Maintenance types	Continued/Maintenance Verbal-Directed (CVD): Sustaining a topic using only verbal language (e.g., answering a question). Continued/Maintenance Non-Verbal-Directed (CND): Using only non-verbal means (e.g., nodding and smiling). Continued/Maintenance Verbal with Non-Verbal (CVN): Using both verbal and non-verbal means simultaneously.
	Disruption and unclassified	Disruption of Maintenance (DIS): Failure of the listener to respond to the prior turn, leading to a conversation breakdown Unclassified/Unknown Behavior (UNK): Utterances not functioning as initiation or maintenance (e.g., private speech).
	Initiation–response ratio	Ratio of child-initiated turns to adult-elicited responses.
	Topic maintenance ratio	Proportion of turns coded as contingent within topic episodes.
Repairs	Type	Self-repair vs. other-initiated repair.
	Successful repair rate	Proportion of repair attempts resolved within two subsequent turns.
Turn-taking	Turns (initiation vs. response)	Each speaker change coded; initiation vs. response labeled.
	Turn-taking productivity	Number of child turns, mean length of utterance (MLU), average words per turn and response latency.
Gestures	Gesture type	Deictic, iconic, beat and metaphoric.
	Gesture phase	Preparation, stroke, hold and retraction.
Multimodal coordination	Gesture–speech alignment	Temporal relation of stroke apex to lexical affiliate: synchronous, preceding, or following.
	Gesture–speech synchrony index	Proportion of lexical affiliates accompanied by a gesture stroke apex within ±500 ms.

###### 2.4.1.4.1 Lexical diversity

Utterances are segmented at the syntactic and prosodic levels, with pauses ≥2 s or a clear topic shift marking new boundaries. Words are segmented using Jieba, with manual correction to ensure accuracy. These transcripts allow for calculation of lexical diversity indices, including type–token ratio (TTR), VOCD-D, and MTLD.

###### 2.4.1.4.2 Communicative acts

Pragmatic functions are coded according to the Inventory of Communicative Acts – Abridged (INCA-A), which covers both communicative intentions and speech acts (see Supplementary File 2) (Agrawal et al., 2024). Each child’s utterance is assigned to an INCA-A category. The distribution of communicative acts across categories provides indices of pragmatic range and balance.

###### 2.4.1.4.3 Initiation and maintenance

Initiation and maintenance are coded at the turn level, distinguishing verbal initiation (IVD), non-verbal initiation (IND), and verbal with non-verbal initiation (IVN), as well as verbal maintenance (CVD), non-verbal maintenance (CND), verbal with non-verbal maintenance (CVN), disruption of maintenance (DIS), and unclassified behavior (UNK). Derived measures include the initiation–response ratio (child-initiated vs. adult-elicited turns) and the topic maintenance ratio (proportion of contingent turns within topic episodes). In this protocol, a turn is operationalized as contingent if it meaningfully builds upon the semantic referents established in the prior speaker’s turn. Specifically, a turn is coded as contingent only if it is both temporally linked and functionally related to the prior proposition (Carolus et al., 2024). Topic episode boundaries are determined by identifying shifts in the continuity of these contingent links; following Bloom and Lahey (1978) and Casillas (2014), a new topic is initiated through the introduction of unlinked referents that do not function as expansions of the previous discourse (Prutting and Kirchner, 1987; Casillas, 2014). For each new topic episode, the “initiator” (child vs. adult) is coded to analyze the child’s active role in discourse management vs. their responsiveness to adult scaffolding.

###### 2.4.1.4.4 Repairs

Repairs are coded by type, distinguishing self-repairs from other-initiated repairs (e.g., requests for repetition, confirmation, explanation, or elaboration). Repair success is defined as resolution within two subsequent turns, and the successful repair rate is derived from these annotations.

###### 2.4.1.4.5 Turn-taking

Conversational turns are marked whenever a speaker change occurs, and each turn is labeled as either an initiation or a response. From these annotations, measures of turn-taking productivity are derived, including the number and proportion of child turns, mean length of utterance (MLU), average words per turn and response latency to the partner’s preceding turn.

###### 2.4.1.4.6 Gestures

Gestures are coded following McNeill’s framework into deictic, iconic, beat and metaphoric types (McNeill, 2005). To ensure data rigor, communicative gestures are strictly distinguished from non-communicative “self-adaptors” (e.g., fidgeting) by verifying the presence of a clear stroke phase and its semantic alignment with speech. Highly ambiguous movements or partial strokes that do not meet these criteria are excluded (McNeill, 1994). Each gesture is segmented into four phases: preparation, stroke, hold and retraction. These codes characterize the form and function of children’s non-verbal communication.

###### 2.4.1.4.7 Multimodal coordination

A dedicated alignment tier is used to annotate the temporal relationship between gestures and speech. Gesture–speech coordination is classified as synchronous, preceding or following the lexical affiliate, which is defined as the specific word or phrase demonstrating the closest semantic correspondence to the gesture’s stroke. Rather than treating all content words as affiliates, this precise mapping focuses on semantically central elements to capture micro-level coordination (Gullberg, 1998). The gesture–speech synchrony index is calculated as the proportion of lexical affiliates accompanied by a gesture stroke apex within ±500 ms. This window is selected to account for the increased temporal variability and coordination delays often observed in developing and neurodivergent populations (Eigsti and Pouw, 2024), providing a more conservative threshold than the ±1,000 ms windows frequently used in early child language acquisition research (Eriksson, 2018).

##### 2.4.1.5 Pilot coding and refinement

Pilot data will be collected from a small group of preschool children to provide the conversational material necessary for method development. Participants for the pilot phase will be recruited from the same sources as the main study (kindergartens and child development centers) to ensure consistent socioeconomic and demographic backgrounds; however, they will serve solely for method development and will not be included in the Phase 2 analysis or compared with the final clinical groups. For this stage, children from the typically developing (TD) group will be selected to establish baseline feasibility and refine coding procedures. Approximately 10–12 children aged 4–6 years will be invited to complete the elicitation tasks, with sessions designed to yield at least 20 min of analyzable conversation and a minimum of 100 child utterances per child. This sample size is sufficient to generate a representative range of conversational phenomena for piloting the transcription and annotation framework.

All pilot recordings will undergo full transcription and multimodal annotation in accordance with the draft coding manual. To evaluate the clarity of definitions and the consistency of coding decisions, approximately 20% of the data will be double-annotated by two independent coders. Inter-rater reliability will be calculated for all major coding categories, with Cohen’s *κ* used for categorical variables (e.g., gesture type and repair category) and intraclass correlation coefficients (ICC) for continuous or rate-based measures (e.g., turn counts and speech–gesture synchrony). Based on established criteria (Landis and Koch, 1977), thresholds of κ ≥ 0.75 and ICC ≥ 0.75 are set to ensure strong agreement. To ensure both stability and consistency, a dual-monitoring approach will be implemented. First, intra-rater reliability will be assessed by having each primary coder re-annotate a randomly selected 10% of their samples after a 4-week interval. Second, to prevent “coder drift” during the ongoing process, quality checks will be performed after every 10 participants. If agreement falls below κ = 0.70, a retraining session will be triggered (Ortiz et al., 2024).

Feasibility will also be monitored during piloting. Specific indicators include task completion rate (target ≥ 90%), usable recording duration (≥20 min per child), and the number of analyzable utterances per task (≥100). These observations will help determine whether the elicitation tasks and recording procedures are practical and engaging for children, and whether adjustments are needed before the full study (see Table 2).

**Table 2:** Feasibility and reliability benchmarks used in pilot coding and refinement.

Indicator	Operational definition	Benchmark
Session completion rate	Percentage of children who complete both elicitation tasks without discontinuation.	≥90%
Usable recording duration	Length of analyzable recording per child (min).	≥20 min
Utterance yield	Number of analyzable child utterances obtained per session.	≥100 utterances
Inter-rater agreement (categorical codes)	Agreement for codes such as topic boundaries and repair types, reported as Cohen’s κ.	κ ≥ 0.75 (good agreement)
Inter-rater agreement (continuous/rate measures)	Agreement for measures such as turn counts or gesture synchrony, reported as ICC (2,1).	ICC ≥ 0.75 (good agreement)
Intra-rater reliability	Stability of coding for 10% of samples re-annotated by the same coder after a four-week interval.	κ ≥ 0.75; ICC ≥ 0.75
Drift monitoring	Proportion of samples re-coded after every 10 participants to check coder drift.	retrain if *κ* < 0.70

The refinement process follows a systematic feedback loop. For example, if the pilot data reveal that certain role-play scenarios result in a task-completion rate below the 90% threshold due to high cognitive demand, the task prompts will be refined by adding more visual scaffolding or simplifying the narrative context. Similarly, if inter-rater reliability for a specific gesture category (e.g., “metaphoric”) falls below the target κ = 0.75, the operational definitions will be refined by incorporating narrower boundary markers and specific exclusionary rules to reduce ambiguity. This iterative step ensures that every adjustment is evidence-based and tailored to the behavioral characteristics of the target population.

The refined coding manual produced through pilot coding will serve as the finalized annotation protocol for Phase 2. This iterative process ensures that the framework is not only theoretically grounded but also practically feasible, reliable across coders and ecologically valid for Mandarin-speaking preschool conversations.

#### 2.4.2 Phase 2: application in DLD and TD groups

In the second phase, the finalized method will be systematically applied to both children with Developmental Language Disorder (DLD) and typically developing (TD) peers, matched for age and gender. This phase aims to compare the conversational and multimodal characteristics across both groups, illustrating the clinical utility of the proposed framework in distinguishing DLD from TD profiles.

##### 2.4.2.1 Sample size

A total of 180 children will be included in this study, with 90 children with DLD and 90 age- and sex-matched typically developing peers. While previous studies investigating conversational abilities and pragmatic language skills in children with DLD typically employed group sizes ranging from 20 to 40 participants per group, which has been sufficient to detect meaningful differences in key outcomes (Osman et al., 2011; Wilder and Redmond, 2022; Winters et al., 2022), we will expand the sample size to 30 participants (30 DLD and 30 TD) per age-stratified group (4-year-old, 5-year-old, and 6-year-old groups). This chosen sample size balances statistical sensitivity with the practical demands of annotating naturalistic language samples, while ensuring sufficient power to detect developmental shifts in multimodal integration.

##### 2.4.2.2 Participants and recruitment

The study will include 180 Mandarin-speaking children stratified into three age groups: 4-year-old (48–59 months), 5-year-old (60–71 months) and 6-year-old (72–83 months) groups. Participants in the DLD group will be recruited through speech and language clinics based on standardized diagnostic criteria. Participants in the DLD group are included if they meet the following conditions: (1) meet the DSM-5 diagnostic criteria for DLD (operationalized via standardized testing and parent interviews regarding functional communicative impact); (2) have Mandarin Chinese as their first language; (3) score below 80 on at least one subscale of the Diagnostic Receptive and Expressive Assessment of Mandarin for Children (DREAM-C) (Liu et al., 2017); (4) have a Full-Scale Intelligence Quotient (FSIQ) above 70 as measured by the Wechsler Intelligence Scale for Children (WISC); and (5) obtain written informed consent from a parent or legal guardian. Children are excluded if they have (1) hearing impairment (failing a formal audiological screening [thresholds > 20 dB HL at 0.5–4 kHz] within the past 12 months); (2) intellectual disability; (3) autism spectrum disorder (ASD); or (4) a history of neurological disorders such as epilepsy, traumatic brain injury or encephalitis.

The TD group will be recruited from mainstream kindergartens and primary schools. Each TD child is individually matched to a child with DLD within their respective age stratum based on age (within a 3-month range) and sex, following a 1:1 matching design. TD children are included if they (1) are within a 3-month age range of the matched child with DLD; (2) have Mandarin Chinese as their first language; and (3) obtain written informed consent from a parent or legal guardian. Children are excluded if they present with any of the following conditions: (1) language disorder; (2) hearing impairment; (3) intellectual disability; (4) autism spectrum disorder (ASD); or (5) a history of neurological disorders such as epilepsy, traumatic brain injury or encephalitis.

Prior to participation, written informed consent will be obtained from parents or legal guardians. Demographic data, developmental history and relevant medical or educational background will be collected through structured caregiver interviews.

##### 2.4.2.3 Measures

Prior to data collection, caregivers will complete a questionnaire on demographics and home environment, including child and family background, developmental history and key aspects of the caregiving context such as shared reading and educational practices.

Children recruited from the outpatient clinic will undergo cognitive and language assessments to determine eligibility for inclusion in the DLD group. Conversational language samples of no less than 20 min are collected from all eligible participants using audio and video recordings.

###### 2.4.2.3.1 Demographic characteristics

Include children’s gender, age, grade, mother’s pregnancy and childbirth history, birth status, parents’ educational level, monthly family income, parents’ occupation, etc.

###### 2.4.2.3.2 Family environment data

Include the frequency of parent–child shared reading, perceived quality of the parent–child relationship, parenting styles and educational practices at home.

###### 2.4.2.3.3 Cognitive assessment

Children’s intellectual functioning is assessed using the Wechsler Preschool and Primary Scale of Intelligence–Fourth Edition (WPPSI-IV) or the Wechsler Intelligence Scale for Children–Fourth Edition (WISC-IV) according to children’s age.

###### 2.4.2.3.4 Language assessment

The Diagnostic Receptive and Expressive Assessment of Mandarin–Comprehensive (DREAM-C) is used to evaluate the language abilities of children in the DLD group. DREAM-C is a standardized diagnostic instrument specifically developed for assessing language development in Mandarin-speaking children aged 2 years 6 months to 7 years 11 months. It provides a comprehensive evaluation of language functioning across five domains: total language, receptive language, expressive language, semantics and syntax (Liu et al., 2017).

###### 2.4.2.3.5 Language sample collection

In Phase 2, the finalized conversational profiling method developed during piloting will be applied to both DLD and TD groups. Data collection will follow the same elicitation tasks and recording procedures described above, ensuring consistency across groups. All language samples will be transcribed and annotated using the refined coding manual, and coders will remain blinded to group membership. Reliability monitoring will continue at the thresholds established in Phase 1. This process will generate the dataset for subsequent analyses.

##### 2.4.2.4 Quality control

All transcription and annotation procedures in Phase 2 will adhere to the feasibility benchmarks (e.g., task completion and recording quality) and reliability standards (*κ*, ICC and drift monitoring) established and refined in the Phase 1 piloting stage. This ensures that only high-quality data that meet the pre-specified inclusion criteria enter the final analysis.

##### 2.4.2.5 Statistical analysis

This study will utilize Excel and Statistical Package for the Social Sciences (SPSS) for statistical analysis.

First, the conversational data will be transcribed using ELAN and exported in CHAT format. The resulting files are then imported into Computerized Language Analysis (CLAN) software, where symbols and formatting are carefully checked for accuracy. Subsequently, several CLAN commands—including FREQ, MLU, MLT, and VOCD—are used to compute the total number of words, utterances, and turns. The software also automatically generates several key measures, including MLU, mean number of words per turn, mean number of utterances per turn, TTR and the lexical diversity D-value.

The “Annotations Statistics” function in ELAN is then used to quantify the frequency of various coded behaviors, including conversation initiation and maintenance, repair, turn-taking, communicative intentions, speech acts and gestures. Finally, the extracted data are organized in Excel and imported into SPSS version 27.0 for further statistical analysis.

###### 2.4.2.5.1 Descriptive statistics

Means, standard deviations, and frequencies will be used to summarize participant demographics, home environment variables, primary language output metrics, conversational measures and gestures.

###### 2.4.2.5.2 Between-group comparisons

To address the first two objectives, independent samples t-tests (or Mann–Whitney U tests for non-normally distributed data) will compare language output metrics, conversational abilities (initiation, topic maintenance, repair and turn-taking) and multimodal features (gesture frequency, gesture–speech alignment patterns) between the DLD and TD groups. Chi-square tests will be used for categorical variables. Group effect sizes will be reported using Cohen’s d or odds ratios, as appropriate.

###### 2.4.2.5.3 Multimodal coordination analysis

Time-stamped annotations from ELAN will be used to compute gesture–speech synchrony indices and the frequency of alignment types (synchronous, preceding and following). Logistic regression or generalized linear mixed models (GLMMs) will test whether gesture–speech alignment predicts conversational coherence and repair success, with random effects included for child and task to account for repeated measures.

###### 2.4.2.5.4 Correlation and regression analyses

Associations between core pragmatic indicators (e.g., topic maintenance ratio, successful repair rate, gesture–speech synchrony) and broader conversational performance measures (e.g., lexical diversity and initiation–response balance) will be examined using Spearman’s rank correlations. Multiple regression models will be applied to identify predictors of conversational success, incorporating language metrics, multimodal measures, and child/family variables.

###### 2.4.2.5.5 Covariate and exploratory analyses

Demographic and family environment variables will be examined as potential covariates in all analyses. Where relevant, exploratory or subgroup analyses will be conducted to assess their influence on conversational performance.

All tests will be two-tailed with significance set at *p* < 0.05. Given the modest sample size, results will be interpreted with attention to statistical power and effect sizes.

## 3 Ethics and data management

This study was reviewed and approved by the Ethics Committee of Nanjing Women and Children’s Healthcare Hospital. All recruited participants agreed to participate and signed a written consent form after receiving written and oral information about the study. Data privacy is maintained by storing raw video recordings and ELAN files on an encrypted, offline server, with access restricted to authorized research personnel. Participants are identified by alphanumeric codes to ensure anonymization across all data tiers. Consistent with research integrity standards, identifiable data will be retained for five years post-study to permit scientific verification, after which all digital files will be permanently purged. Parents are informed of their right to withdraw consent at any time; in such instances, all relevant video and transcription files will be immediately and permanently deleted.

## 4 Discussion

This protocol describes the development and application of a multimodal framework for profiling conversational abilities in Mandarin-speaking preschool children. The approach combines ecologically valid elicitation tasks with a detailed transcription and annotation system that integrates verbal, non-verbal and gesture–speech synchrony measures. By including feasibility indicators, reliability benchmarks, and an iterative piloting stage, the protocol offers a reproducible methodological foundation. This framework serves as a methodological proposition to validate the feasibility and suitability of multimodal profiling within the Mandarin-speaking population.

A key strength of this protocol lies in its integration of multimodal data. Whereas the majority of studies on DLD focus exclusively on verbal transcripts, this framework captures both linguistic and gestural behaviors, as well as their temporal alignment. This allows researchers to explore pragmatic dimensions of conversation that are otherwise overlooked, including the role of gestures in topic maintenance, clarification, and repair. In addition, the use of structured role-play alongside free conversation tasks ensures that conversational samples are both naturalistic and sufficiently varied to elicit a wide range of pragmatic behaviors.

Despite these strengths, several limitations must be acknowledged. The cross-sectional design precludes examination of developmental trajectories. Specifically, a single-point assessment cannot definitively distinguish whether the identified multimodal differences are stable trait markers or transient delays. However, theoretical evidence suggests that certain measures proposed in this protocol represent promising, theoretically motivated candidate indicators of DLD. For instance, empirical findings indicate that gesture quality—encompassing formal accuracy and motor precision—serves as a persistent neurodevelopmental marker of DLD, whereas gesture quantity may merely reflect transient compensatory strategies. While children with DLD often maintain typical gesture rates, their significant deficits in gesture execution represent a stable trait that distinguishes them from typically developing peers across different developmental stages (Wray et al., 2017). Furthermore, the temporal integrity of gesture–speech synchrony (e.g., our ±500 ms window) is considered a foundational component of the language production architecture (Kopp et al., 2013); deficits in such cross-modal integration could serve as potential indicators of underlying temporal processing impairments rather than simple developmental lags, though longitudinal data will be essential to establish their stability over time. In contrast, structural linguistic indices such as lexical diversity (TTR) may represent more transient differences that are susceptible to age-related growth. Furthermore, while the age-stratified design (*N* = 180) enhances the potential for identifying developmental trends, the inherent complexity of high-dimensional, multimodal data suggests that statistical power for certain subtle interaction effects should be interpreted with caution. Although generalized linear mixed models (GLMMs) should be employed to refine estimation precision by accounting for individual variability, the current framework should be regarded as an exploratory foundation for future large-scale power analyses. Furthermore, although semi-automated support is incorporated, much of the annotation process remains labor-intensive, potentially limiting scalability in larger studies. Consequently, the current study focuses on identifying diagnostic indicators within a specific developmental window (ages 4–6), but its utility for predicting long-term clinical prognosis or tracking intervention responsiveness remains to be validated. Finally, the present protocol is restricted to Mandarin-speaking preschoolers. While the foundational principles may be informative for other contexts, future empirical testing is required to confirm whether these specific Mandarin-adapted measures remain valid across different linguistic environments.

Future research should extend this protocol in several directions. Longitudinal studies may apply the framework to track changes in conversational abilities over time, helping to distinguish between “trait” and “state” markers in multimodal integration. While clinical researchers may adapt the protocol to evaluate targeted interventions, longitudinal data will be essential to establish the responsiveness of these measures—specifically whether improvements in synchrony or latency serve as sensitive indices of progress. The integration of more advanced automated tools for speech recognition and gesture tracking could further improve scalability and reduce coder burden. Future validation in other language families could help determine the universality of the indicators developed here, though such a study lies beyond the scope of this initial Mandarin-focused protocol. Moreover, current observations are limited to child–adult dyads; future iterations should incorporate child–peer interactions to capture pragmatic difficulties in less-supported, naturalistic social contexts.

Beyond these future directions, a practical next step is to establish a framework to ensure procedural consistency and data comparability among researchers applying this protocol within Mandarin-speaking contexts. To facilitate dissemination, this protocol prioritizes procedural transparency by providing comprehensive operational definitions and ELAN tier specifications within the manuscript (see Table 1), allowing independent groups to adapt the coding environment to their specific linguistic and cultural contexts. For data harmonization, researchers are encouraged to use standardized data-output configurations, such as structured tab-delimited exports with consistent tier-to-column mapping (e.g., unified columns for “Onset_Time,” “Speech_Content,” and “Gesture_Type”). This ensures that findings from independent studies can be systematically aligned and collated. Furthermore, the replicability of the protocol is defined by its methodological stability: first, standardized rater training, where independent investigators conduct internal reliability trials to achieve high inter-rater agreement (Cohen’s *κ* > 0.75); and second, the consistency of developmental patterns, where the protocol is expected to identify stable performance differences between TD and DLD groups across different samples. Such standardization ensures that individual studies contribute to a more integrated and reliable evidence base for DLD research within this linguistic environment.

In the longer term, this protocol is envisioned to serve as a methodological blueprint that may eventually be adopted across diverse research and clinical settings. By adhering to the shared data configurations and technical benchmarks established here, future studies can ensure effective harmonization, enabling systematic comparison and, potentially, the collation of findings into a broader evidence base. Furthermore, the replicability of this framework should be rigorously evaluated through multi-center collaborative studies within and beyond Mandarin-speaking regions. Such widespread implementation will help clarify how these candidate indicators can be scaled into a validated clinical tool, ultimately supporting the design of more effective, evidence-based interventions for children with DLD.
