# Supplementary material for: A methodological protocol for multimodal profiling of conversational abilities in mandarin-speaking children with and without developmental language disorder
Source: Front Psychol. 2026 May 5;17:1704308. doi: 10.3389/fpsyg.2026.1704308 (PMC13183633; doi:10.3389/fpsyg.2026.1704308)
Supplement: Supplementary file 1 [file Table_1.DOCX]

**Semi-structured free conversation**

10-15 minutes

****Introduction:**** ****"Hello there, look, there's a little dog here. His name is Archie. He wants to be your friend and have a chat with you. He will ask you some questions, and you can ask him questions too. Are you ready? Let's begin now!"****

****Reference Topics****

| **Module 1：family** | | **Child Information** |
| --- | --- | --- |
| Talk about siblings | Topic 1. How old are you?   1. When is your birthday? 2. What do you do on your birthday? | Name：  Gender：  Age：  Date of Birth： |
|  | Topic 2. Do you have any siblings?   1. What are their names? 2. How old are they? 3. What do you like to do with your siblings? | Siblings: (Yes/No & details) |
| Talk about pets | Topic 3. Do you have any pets at home?   1. What kind of pet? 2. Tell me how you take care of he/she? 3. My kitten can..., can your pet do that?？ | Pet Type:  Pet Name: |
| ****Module 2: Favorite Things to Do**** | | ****Child Information**** |
| Tell the child how you spend your free time. Talk about games you like and how you play them. | Topic 4. What do you like to do most at home?   1. Do you like playing games with xxx[e.g., mom, dad, sibling]? 2. How do you play it? | Favorite Game: |
| Talk to the child about your favorite thing about kindergarten | Topic 5. What do you like most about kindergarten?   1. What do you usually do in kindergarten? 2. Tell me why you like [Thing from A] at kindergarten. | Grade/Year: |
| Talk about favorite toys | Topic 6. What is your favorite toy?   1. Who bought this toy for you? 2. Who do you usually play with? 3. How do you play with this toy? | Favorite Toy: |
| ****Module 3: Favorite Cartoon**** | | ****Child Information**** |
| Talk about your favorite cartoon and why you like it | Topic 7. Do you have a favorite cartoon?   1. What is this cartoon about? 2. Who is your favorite character? Why? | Favorite Cartoon: |

**"Going Shopping" Role-Playing Game**

10-15 minutes

****"Hey there, look, this is a supermarket. I'd like to play a shopping game with you. I'll be the shop assistant first, and you can be the little customer, okay? Later we can switch roles."**** After segment 5 is finished, say to the child: ****"Now let's switch. You be the shop assistant, and I'll be the customer, okay?"****

****Examples are as follows：****

| 1 | Examiner: "Welcome to my supermarket! I have lots of things here, come and see what we have." |
| --- | --- |
| 2 | Examiner: "What would you like to buy?" |
| 3 | Examiner: "Would you like to buy anything else?" (Show multiple toys) |
| 4 | Examiner: "Let's go to the checkout to pay." (Wait for the child to initiate) |
| 5 | Examiner: "That will be 10 kuai (RMB)." (Wait for the child's reaction) |
| 6 | Examiner: Wait for the child to initiate conversation: "What would you like to buy?" |
| 7 | Examiner: "Hello, I'd like to buy some fruit. What kinds of fruit do you have here?" (Wait for the child's reaction) |
| 8 | Examiner: "I also want to buy xxx." (Wait for the child's reaction) |
| 9 | Examiner: "How much do these cost?" (Wait for the child's reaction) |
| 10 | Examiner: "That's too expensive, can it be cheaper?" (Wait for the child's reaction) |
