# Supplementary material for: A methodological protocol for multimodal profiling of conversational abilities in mandarin-speaking children with and without developmental language disorder
Source: Front Psychol. 2026 May 5;17:1704308. doi: 10.3389/fpsyg.2026.1704308 (PMC13183633; doi:10.3389/fpsyg.2026.1704308)
Supplement: Supplementary file 2 [file Table_2.DOCX]

**Inventory of Communicative Acts–Abridged (INCA-A)**

**Types of communicative intentions**

| Code | Content | Explanation |
| --- | --- | --- |
| NCS | Negotiate co-presence and separation | To manage the transition. |
| NMA | Negotiate mutual attention and proximity | To establish mutual attentiveness and proximity or withdrawal. |
| SAT | Showing attentiveness | To demonstrate that speaker is paying attention to hearer. |
| DHA | Directing hearer's attention | To achieve joint focus of attention by directing hearer's attention to objects, persons and events in the environment. |
| DJF | Discussing a joint focus of attention | To hold a conversation about something in the environment that both participants are attending to, e.g. objects; persons; ongoing actions of hearer and speaker; ongoing events. |
| DRP | Discussing the related-to-present | To discuss non-observable attributes of objects or persons present in the environment or to discuss past or future events related to those referents. |
| DRE | Discussing a recent event | To hold a conversation about immediately past actions and events |
| DNP | Discussing the non-present | To hold a conversation about topics which are not observable in the environment, e.g. past and future events and actions, distant objects and persons, abstract matters. (Excluding conversations about hearer's and speaker's inner states.) |
| DFW | Discussing the fantasy world | To hold a conversation within fantasy play |
| DHS | Discussing hearer's thoughts and feelings | To hold a conversation about hearer's non-observable thoughts and feelings. |
| DSS | Discussing speaker's thoughts and feelings | To hold a conversation about speaker's non-observable thoughts and feelings. |
| PSS | Negotiating possession of objects | To determine or discuss who is the possessor of an object. |
| NIA | Negotiating the immediate activity | To negotiate the initiation, continuation, ending and stopping of activities and acts; to direct hearer's and speaker's acts; to allocate roles, moves and turns in joint activities; to evaluate speaker's and hearer's acts as correct or incorrect; or as desirable or undesirable. |
| NFA | Negotiating an activity in the future | To negotiate actions and activities in the far future. |
| PRO | Performing verbal moves in an activity | To perform moves in a game or other activity by uttering the appropriate verbal forms. |
| MRK | Marking | To express socially expected sentiments on specific occasions such as thanking. |
| CMO | Comforting | To comfort hearer, to express sympathy for misfortune. |
| DCC | Discussing clarification of verbal communication | To discuss clarification of hearer's ambiguous verbal communication, or a confirmation of speaker's understanding of it. |
| DCA | Discussing clarification of action | To discuss clarification of hearer's nonverbal communicative acts. |
| TXT | Read written text | To read or recite written text aloud. |
| NIN | Non-interactive speech | Speaker engages in private speech or produces utterances which are clearly not addressed to present hearer. |
| OOO | Unintelligible utterances | Unknown function |
| YYY | Uninterpretable utterances | Unknown function |

**Speech acts**

| ****Directives and responses**** | |
| --- | --- |
| RP | Request/propose/suggest action for hearer. Proposed action might also involve speaker. |
| RQ | Yes/no question about hearer's wishes and intentions which functions as a suggestion. |
| DR | Dare and challenge hearer to perform action. |
| WD | Warn of danger. |
| CL | Call attention of hearer by name or by substitute exclamations. |
| SS | Signal to start performing an act, e.g., to run or roll a ball. Pace performance of acts by hearer. |
| AD | Agree to do; agree to carry out act requested or proposed by other. |
| AL | Agree to do for the last time. |
| RD | efuse to do; refuse to carry out act requested or proposed by other. Including refusals by giving excuses and reasons for noncompliance. |
| CS | Counter-suggestion; an indirect refusal. |
| GI | Give in: accept other's insistence or refusal. |
| AC | Answer calls; show attentiveness to communications. |
| GR | Give reasons, justify requirement, refusal or prohibition of an act. |
| ****Speech elicitations and responses**** | |
| EI | Elicit imitation of word or sentence by explicit command. |
| EC | Elicit completion of word or sentence. |
| EX | Elicit completion of rote-learned text. |
| RT | Repeat/imitate other's utterance. |
| SC | Complete statement or other utterance in compliance with request eliciting completion. |
| CX | Complete text if so demanded. |
| EA | Elicit animal or sound imitation |
| ****Commitments and responses**** | |
| SI | State intent to carry out act by speaker; describe speaker's own ongoing activity. |
| FP | Ask for permission to carry out act by speaker. |
| PD | Promise. |
| TD | Threaten to do. |
| PA | Permit hearer to perform act. |
| PF | Prohibit/forbid hearer to perform act. |
| ****Declarations and responses**** | |
| DC | Declare; create a new state of affairs by declaration. |
| DP | Declare (phantasy); create make-believe reality by declaration. |
| YD | Agree to a declaration. |
| ND | Disagree with a declaration. |
| ****Markings and responses**** | |
| MK | Mark occurrence of event (ie thank, greet, apologize, congratulate, mark completion of action, etc.) |
| TO | Mark transfer of object to hearer. |
| CM | Commiserate, express sympathy for hearer's distress. |
| EM | Exclaim in distress, pain. |
| EN | Endearment; express positive emotion. |
| ES | Exclaim in surprise; express surprise. |
| XA | Exhibit attentiveness to hearer. |
| ****Statements and responses**** | |
| ST | State; make a declarative statement. |
| AP | Agree with proposition expressed by previous speaker. |
| DW | Disagree with proposition expressed by previous speaker. |
| WS | Express a wish. |
| CN | Count. |
| ****Questions and responses**** | |
| QN | Wh-question; ask a product-question. |
| YQ | Yes/no question; ask a yes/no question. |
| TQ | Two-alternative yes/no question. |
| EQ | Elicit question |
| AQ | Object by re-asking a question. |
| SA | Answer a wh-question by a statement. |
| AA | Answer in the affirmative to yes/no question. |
| AN | Answer in the negative to yes/no question. |
| QA | Answer a question with a wh-question. |
| YA | Answer a question with a yes/no question. |
| TA | Answer a two-alternative question. |
| NA | Non-satisfying answer to question. |
| RA | Refuse to answer. |
| DK | Answer "don't know". |
| ****Performances**** | |
| PR | Perform verbal move in game. |
| TX | Read written text aloud. |
| ****Evaluations**** | |
| PM | Praise for motor acts, i.e. nonverbal behavior. |
| ET | Exclaim in enthusiasm; express enthusiasm for hearer's performance. |
| CR | Criticize; point out error in nonverbal act. |
| AB | Approve of appropriate behaviour. Express positive evaluation of hearer's or speaker's acts. |
| DS | Disapprove, scold, protest disruptive behavior. Express negative evaluation of hearer's or speaker's behaviour as inappropriate. |
| ED | Exclaim in disapproval. |
| ****Demands for clarification**** | |
| RR | Request to repeat utterance. |
| ****Text editing**** | |
| CT | Correct; provide correct verbal form in place of erroneous one. |
| ****Vocalizations**** | |
| YY | Word babble; utter a word-like utterance without clear function. |
| OO | Idiosyncratic words |
